# Novel Insights Into the Sulfated Glucuronic Acid-Based Anti-SARS-CoV-2 Mechanism of Exopolysaccharides From Halophilic Archaeon *Haloarcula hispanica*

**DOI:** 10.3389/fchem.2022.871509

**Published:** 2022-04-27

**Authors:** Yueqiang Xu, Yan Li, Xin You, Caixia Pei, Zhuo Wang, Siming Jiao, Xin Zhao, Xuan Lin, Yang Lü, Cheng Jin, George Fu Gao, Jianjun Li, Qi Wang, Yuguang Du

**Affiliations:** ^1^ State Key Laboratory of Biochemical Engineering, National Engineering Research Center for Biotechnology (Beijing), Key Laboratory of Biopharmaceutical Production & Formulation Engineering, PLA, Institute of Processing and Engineering, Chinese Academy of Sciences, Beijing, China; ^2^ CAS Key Laboratory of Pathogenic Microbiology and Immunology, Institute of Microbiology, Chinese Academy of Sciences, Beijing, China; ^3^ Lung Cancer Translational Medicine Center, The Second Affiliated Hospital of Dalian Medical University, Dalian, China; ^4^ State Key Laboratory of Mycology, Institute of Microbiology, Chinese Academy of Sciences, Beijing, China

**Keywords:** sulfated glucuronic acid, SARS-CoV-2, exopolysaccharide, archaea, *Haloarcula hispanica*

## Abstract

The pandemic caused by SARS-CoV-2 is the most widely spread disease in the 21st century. Due to the continuous emergence of variants across the world, it is necessary to expand our understanding of host–virus interactions and explore new agents against SARS-CoV-2. In this study, it was found exopolysaccharides (EPSs) from halophilic archaeon *Haloarcula hispanica* ATCC33960 can bind to the spike protein of SARS-CoV-2 with the binding constant K_D_ of 2.23 nM, block the binding of spike protein to Vero E6 and bronchial epithelial BEAS-2B cells, and inhibit pseudovirus infection. However, EPSs from the gene deletion mutant *△HAH_1206* almost completely lost the antiviral activity against SARS-CoV-2. A significant reduction of glucuronic acid (GlcA) and the sulfation level in EPSs of *△HAH_1206* was clearly observed. Our results indicated that sulfated GlcA in EPSs is possible for a main structural unit in their inhibition of binding of SARS-CoV-2 to host cells, which would provide a novel antiviral mechanism and a guide for designing new agents against SARS-CoV-2.

## Introduction

SARS-CoV-2 represents one of the most fast-spreading viruses in the 21st century (Tan et al., 2020; Wang C et al., 2020), and the pandemic has swept across the world. More than 300 million people were infected, and five million were killed by the virus. Several variants of the virus have been designated as variants of concerns (VOCs), including B.1.1.7 (alpha), B.1.351 (beta), P.1 (gamma), B.1.617.2 (delta), and B.1.1.529 (omicron). Extensive studies have focused on vaccines neutralizing antibodies and antiviral chemical compounds. However, these efforts were challenged by the more virulent and easily transmitted SARS-CoV-2 variants. Epecially for the variant of omicron, both vaccines and neutralizing antibodies displayed reduced neutralizing titers (Cao et al., 2021; Lu et al., 2021). In addition to the urgent demand of preventive and therapeutic strategies, it is also necessary to deeply understand the interaction between virus and host cells in nature.

The receptor-binding domain (RBD) of spike protein of SARS-CoV-2 can bind to angiotensin-converting enzyme 2 (ACE2) on the surface of host cells specifically, then the activated proteases such as furin, transmembrane serine protease 2 (TMPRSS2), or cathepsin L cleave the spike protein, and finally the HR1 and HR2 regions in the S2 subunit interact with the cell membrane to mediate fusion, resulting in the release of the viral genome into the cytoplasm (Shang et al., 2020; Saied et al., 2021). The 3D structures of SARS-CoV-2 virus, S protein, and human ACE2 (hACE2) have been determined (Walls et al., 2020; Wang Q et al., 2020). The spike protein of SARS-CoV-2 is heavily glycosylated with 22 *N*-glycosylation sites and 17 *O*-glycosylation sites (Shajahan et al., 2020; Tian et al., 2021), whereas hACE2 employs seven *N*-glycosylation sites and one *O*-glycosylation site (Shajahan et al., 2021). In addition to ACE2, there might be multiple receptors or co-receptors in host cells for SARS-CoV-2 infection, including heparan sulfate (HS) on the host cell surface, and immune mannose receptors of DC-SIGN, L-SIGN, MGL, Siglec-9, and Siglec-10 in cells can also bind with the spike protein (Chiodo et al., 2020; Clausen et al., 2020; Gao et al., 2020). Therefore, an attractive approach to fight against SARS-CoV-2 is to block or interfere with virus attachment and binding to host cells.

Glycans are one of the most important molecules in cells, which play critical roles in virus assembly, attachment, recognition, entry, and immune escape (Watanabe et al., 2019). Viruses can employ glycans as receptors to infect hosts, such as human influenza A viruses recognizing α2, 6-linked sialic acid, and avian influenza A viruses, showing preference for α2, 3-linked sialic acid (Kumlin et al., 2008; Shi et al., 2014; Li et al., 2017). Furthermore, HCoV-OC43, HCoV-HKU1, BCoV, and PHEV can use 9-*O*-acetyl-sialic acid as a receptor (Tortorici et al., 2019). The fact that 9-*O*-acetyl-sialic acid can prevent MERS-CoV from binding to host cells means “glycan inhibitors” might be ideal candidate drugs to fight against virus infection (Li et al., 2017). Several teams have reported that glycans can be used as anti-SARS-CoV-2 agents, such as marine sulfated polysaccharides (Jin et al., 2020; Kwon et al., 2020; Song et al., 2020; Dwivedi et al., 2021; Zhang et al., 2022) and HS (Clausen et al., 2020; Kim et al., 2020; Hao et al., 2021). HS can bind to S protein of SARS-CoV-2 and block binding of the spike protein to hACE2 and can impede the infection by pseudovirus and authentic SARS-CoV-2. HS possesses broad-spectrum activities against a multitude of distinct viruses, including flaviviruses, herpes, influenza, HIV, and Coronaviridae. Recent studies had shown that HS can inhibit the invasion of SARS-CoV-2 depending on its chain length and sulfation pattern (Kim et al., 2020; Mycroft-West et al., 2020; Hao et al., 2021; Liu et al., 2021). For instance, *N*-desulfated HP, 2-*O*-desulfated HP, and 6-*O*-desulfated HP were unable to compete with immobilized HP for binding to SARS-CoV-2 (Kim et al., 2020), while an octasaccharide composed of IdoA2S-GlcNS6S can inhibit spike–heparin interaction with an IC_50_ of 38 nM, and Tris HS hexasaccharide [GlcA (2S)-GlcNS (6S)] also can bind to the trimeric spike protein of SARS-CoV-2 (Liu et al., 2021). Therefore, it is important to elucidate the critical structures responsible for antiviral activity in glycans.

Archaea are one of the most primitive organisms on the Earth and usually live in extreme environments such as saline lakes, Antarctic ecosystems, geothermal springs, and deep sea. Exopolysaccharides (EPSs) from extremophiles can be applied in food, pharmaceutical, and cosmetics industries (Nicolaus et al., 2010). *Haloarcula hispanica* ATCC33960 is an extremely halophilic archaeon isolated from a solar saltern in Spain, which can produce sulfated EPSs (Lü et al., 2017). In this study, our results showed that EPSs from *H. hispanica* ATCC33960 can bind to the spike protein of SARS-CoV-2 inhibit the binding of spike protein to Vero-E6 and bronchial epithelial BEAS-2B cells, and impede the infection of SARS-CoV-2 pseudovirus. As far as we know this is the first discovery that EPSs from archaea can inhibit SARS-CoV-2 infection *in vitro*. Further analysis showed that the GlcA content and the sulfation level of EPSs play essential roles in anti-SARS-CoV-2 activity.

## Materials and Methods

### Strain Culture and Exopolysaccharides Preparation

*Haloarcula hispanica* ATGG33960 was cultured in AS-168 medium to late stationary phase (5 g/L Bacto casamino acids, 5 g/L Bacto yeast extract, 1 g/L sodium glutamate, 3 g/L trisodium citrate, 20 g/L MgSO_4_.7H_2_O, 2 g/L KCl, 200 g/L NaCl, 50 mg/L FeSO_4_.7H_2_O, 0.36 mg/L MnCl_2_.4H_2_O, and pH 7.0). EPSs were first precipitated from the supernatant by 4-fold volume of ethanol and then dialyzed against water. The dialyzed solution was treated with Benzonase nuclease and protease K subsequently at 37°C for 12 h. After concentrated with the 100 kDa ultrafiltration membrane, the EPSs solution was lyophilized. Crude EPSs were further sequentially purified by a DEAE-Sepharose Fast Flow and Sephacryl S-400/HR column as described. The concentration of EPSs was measured by the phenol–sulfuric acid method and determined at A490 (Lü et al., 2017).

### Homogeneity and Molecular Weight

For molecular weight (MW) measurement of EPSs, the samples were analyzed by high-performance gel permeation chromatography (HPGPC) with a TSK GEL GMPWXL column, and the polysaccharides were eluted with a mobile phase containing ddH_2_O at a flow rate of 0.5 mL/min and detected by using an evaporative light-scattering detector (ELSD).

### Sulfate Content Comparison

To evaluate the sulfate content of the polysaccharides, 10 μg of EPSs was run in 7.5% (w/v) SDS-PAGE, then the gel was stained with 0.5% (w/v) methylene blue in 3% (v/v) acetic acid, and SO_4_^2−^ in EPSs can be stained with methylene blue (Lü et al., 2017).

### Monosaccharide Composition Analysis

For monosaccharide analysis, EPSs (5 mg) were hydrolyzed in 2 M trifluoroacetic acid at 120°C for 2 h, and then the solution was evaporated to dryness by using a rotary evaporator after adding 2-fold volume of methanol. Hydrolyzed EPSs were dissolved in 1 mL ddH_2_O, and then the samples were analyzed in HPAEC-PAD with a CarboPac PA-10 column. For analysis of neutral sugars, the elution condition is 18 mM NaOH at a flow rate of 1.0 mL/min, while the acidic sugars were analyzed by 100 mM NaOH and 100 mM CH_3_COONa at a flow rate of 1.0 mL/min. Mannose (Man), galactose (Gal), glucose (Glc), D-glucuronic acid (GlcA), and D-galacturonic acid (GalA) were used as standards.

### Binding Assay of S Protein and Exopolysaccharides

The recombinant spike RBD protein of SARS-CoV-2 expressed in HEK-293 cells was purchased from BioRobust (Shenzhen, China). The binding of glycans to RBD of spike protein was first evaluated by Monolith NT.115 (Nanotemper): 50 μg of RBD was labeled with a Monolith RED-NHS protein Labeling Kit, then a series of EPSs solutions of *H. hispanica* were prepared by a 2-fold serial-dilution method, and the binding affinity between RBD protein and EPSs was evaluated in Monolith NT.115 after co-incubated for 10 min. When the binding capability of glycan was observed from Monolith NT.115, the binding kinetics between glycan and the RBD protein was further determined by biolayer interferometry (BLI)-based assay with Octet R8 (Sartorius): The Ni-NTA sensor was coated with 5 μg/mL RBD for 10 min, then the EPSs solution was diluted by 2-fold with buffer [10 mM PBS +0.02% Tween 20 (w/w)], and the diluted EPSs solution was incubated with the sensors coated with the RBD protein for 2 min. After dissociated for another 2 min, the binding constant K_D_ between EPSs and RBD was measured.

### Cell Culture and Cell Viability Assay

The human bronchial epithelial BEAS-2B cells were cultured in RPMI 1640 with L-glutamine (Corning, 10-040-CV) supplemented with 10% FBS, 200 mg/mL streptomycin and 200 IU/mL penicillin at 37°C, 5% CO_2_. The African green monkey kidney Vero E6 cells were maintained in DMEM (Gibco, 11965092) supplemented with 10% fetal bovine sera (FBS), 200 mg/mL streptomycin, and 200 IU/mL penicillin at 37°C, 5% CO_2_. For cell viability assay, the cells were seeded in a 96-well plate with 1 × 10^4^ cells/well and cultured for 24 h, then the supernatants were discarded, and 150 μl of serial-diluted EPSs in culture medium was added to the cells and incubated for another 24 h. Subsequently, 15 μl of MTT [3-(4,5-dimethyl-2-thiazolyl)-2,5-diphenyl-2-H-tetrazolium bromide, 5 mg/mL] was added to each well and incubated for 4 h, then the supernatants were discarded and 100 μl of DMSO (dimethyl sulfoxide) was added to dissolve the purple precipitate. Finally, the 96-well plate was scanned with Infinite M200 Pro (TECAN) at 405 nm. Data were expressed as the means ± standard errors of the means (SEM). *p* values were analyzed by unpaired *t* test with GraphPad 5.

### Cell-Binding Assay Against S Protein

The cells of BEAS-2B and Vero E6 were seeded into 24-well glass bottom plates (Cellvis) with 1 × 10^5^ cells/well and cultured for 48 h. The cells were washed with PBS three times, and then fixed with 4% (w/v) of paraformaldehyde (PFA). Then, the cells were incubated with 200 μl solution containing EPSs (2 μg/well) and RBD of spike protein (1 μg/well) for 2 h at 37°C. The RBD protein binding to the cells can be detected by SARS-CoV-2 spike-neutralizing antibody, mouse mAb (SinoBiological, 40592-MM57), and Alexa Fluor 488^TM^ goat anti-mouse IgG (H + L) (Invitrogen, A-11001). The nuclear DNA of cells was stained using 4’,6-diamidino-2-phenylindole (DAPI, 1 μg/mL). Images were captured with a Leica TCS SP8 STED confocal microscope, and data were analyzed using LAS X software (Leica).

### Preparation of Pseudotyped Virus and Neutralization Assay

The construction of VSV-ΔG-GFP-based SARS-CoV-2 pseudotyped virus was mentioned in previous work with slight modifications (Zhang Z et al., 2021; Zhao et al., 2021). The codon-optimized wild-type SARS-CoV-2 (Wuhan-1 reference strain) was constructed into the pCAGGS vector. The construct (30 μg) was transfected into HEK 293T cells. VSV-ΔG-G-GFP pseudovirus was added 24 h after the transfection and removed after 1-h incubation. Media were replaced with fresh complete DMEM medium supplemented with anti-VSV-G antibody (I1HybridomaATCC® CRL2700™). Supernatants were collected after another 30-h incubation, passed through a 0.45-μm filter (Millipore, SLHP033RB), aliquoted, and stored at −80°C.

Neutralization was measured by the reduction in GFP expression as described previously (Zhang S et al., 2021). One day before neutralization assay, Vero E6 cells were seeded into 48-well plates with 1 × 10^5^ cells/well and incubated at 37°C. Pseudovirus was incubated with 3-fold serially diluted EPSs for 1 h in advance, together with the virus control and cell control. Then, pseudovirus was transferred to pre-plated Vero E6 cells washed by fresh DMEM without FBS, followed by incubation at 37°C for 24 h. After lysed by trypsin, the GFP positive cells were measured with an FACSCanto II flow cytometer (BD Biosciences, United States).

## Results

### Binding of Exopolysaccharides With the Receptor-Binding Domain of Spike Protein

It has been found that several sulfated polysaccharides could bind with RBD of spike protein including HS, fucoidan, carrageenan, and sulfated polysaccharide from sea cucumber, leading to their interference with binding of RBD to ACE2 at different extents (Clausen et al., 2020; Kim et al., 2020; Song et al., 2020; Hao et al., 2021). Therefore, sulfated polysaccharides were potential candidates against SARS-CoV-2. EPSs from *H. hispanica* were sulfated too, so their binding with RBD was investigated.

**FIGURE 1:**
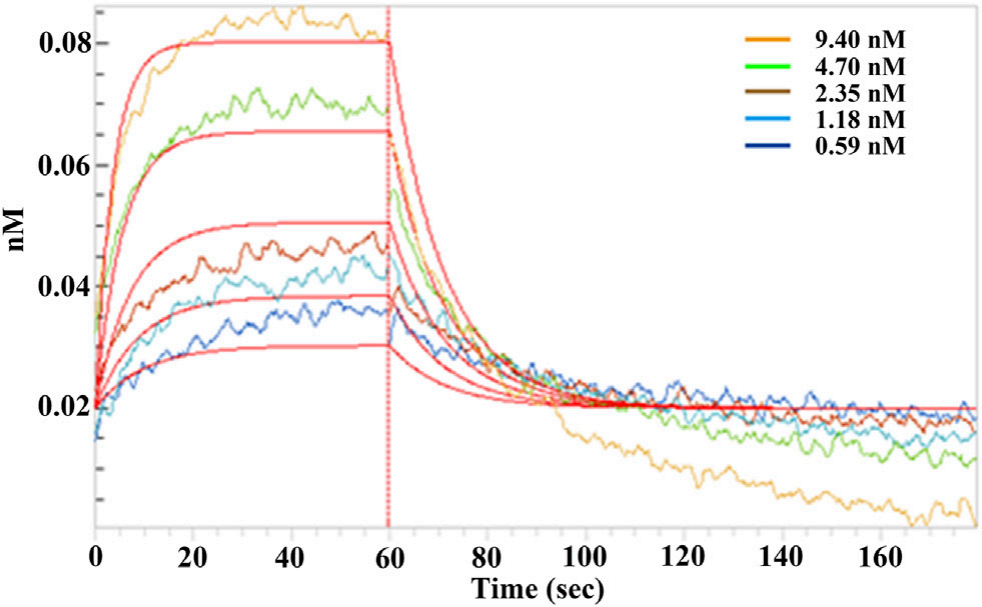
Binding affinity assay between SARS-CoV-2 RBD and EPSs from *H*. *hispanica*. Serially diluted EPSs solutions were incubated with RBD (50 μg), which was coated on a Ni-NTA sensor for 2 min, and the affinity kinetics were analyzed after being dissociated for another 2 min. The binding kinetics between EPSs and RBD were determined by using the biolayer interferometry (BLI) method.

Binding of EPSs from *H. hispanica* to SARS-CoV-2 RBD protein was first analyzed by Microscale thermophoresis (MST). Noticeably, EPSs can bind with RBD protein well (Supplementary Figure S1). The binding kinetics between EPSs and RBD of spike protein were further determined by biolayer interferometry (BLI)-based assay with Octet R8 (Sartorius). The result showed that EPSs from *H. hispanica* can bind to RBD with high affinity with the calculated K_D_ as 2.23 × 10^−9^ M (Figure 1). EPSs from *H. hispanica* displayed good affinity to the RBD protein of SARS-CoV-2.

### Cell Viability Assay

In order to assess the toxicity of EPSs from wild-type of *H. hispanica* and *△HAH_1206*, the cell viabilities were checked after EPSs were incubated with BEAS-2B or Vero E6 cells. The results showed the viabilities of BEAS-2B and Vero E6 cells were affected when the concentrations of EPSs from WT exceeded 12.5 μg/mL (Supplementary Figure S2A). But for EPSs from *△HAH_1206*, the viabilities of BEAS-2B and Vero E6 cells seemed not affected by serially diluted EPSs, only Vero E6 cells were slightly affected at EPSs concentration of l00 μg/mL, which meant that EPSs from *△HAH_1206* nearly lost toxicity. Therefore, the EPSs concentration was set below 12.5 μg/mL in following experiments.

### Interference of Exopolysaccharides With Binding of the Receptor-Binding Domain to Cells

**FIGURE 2:**
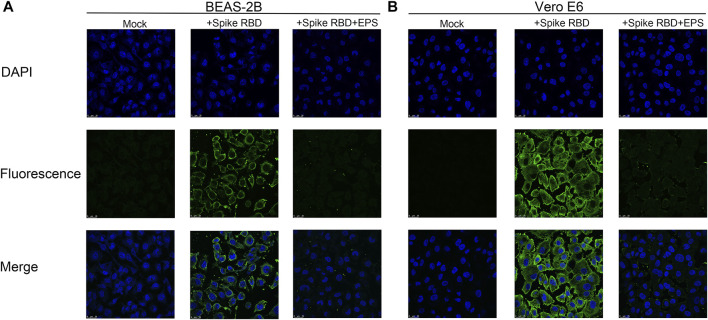
Immunofluorescence assay of EPSs from *H. hispanica*-blocking RBD to bind with BEAS-2B and Vero E6. **(A)** Binding of RBD to BEAS-2B cells inhibited by EPSs from *H*. *hispanica*. **(B)** Binding of RBD to Vero E6 cells inhibited by EPSs from *H. hispanica*. **Mock**: cells detected in the absence of RBD and EPSs; + **Spike RBD**: cells detected in the presence of RBD (1 μg/well); and + **Spike RBD** + **EPSs**: cells detected in the presence of RBD (1 μg/well) and EPSs (2 μg/well). All wells were detected by immunofluorescence using SARS-CoV-2 (2019-nCoV) spike-neutralizing antibody and Alexa Fluor 488 goat anti-mouse IgG (H + L) by using a confocal microscope. The fluorescence signals were captured with an FITC channel, and the nuclear DNAs of cell were stained with DAPI.

Interference of EPSs with the binding of RBD to cells was further investigated *via* immunofluorescence. It was observed that RBD can bind to BEAS-2B and Vero E6 cells. However, the signals from Alexa Fluor 488 were blurry when EPSs were incubated with RBD in advance. These results clearly demonstrated that EPSs could block the interaction between RBD and cells expressing hACE2 (Figure 2).

### Anti-Infection of Pseudovirus by Exopolysaccharides

**FIGURE 3:**
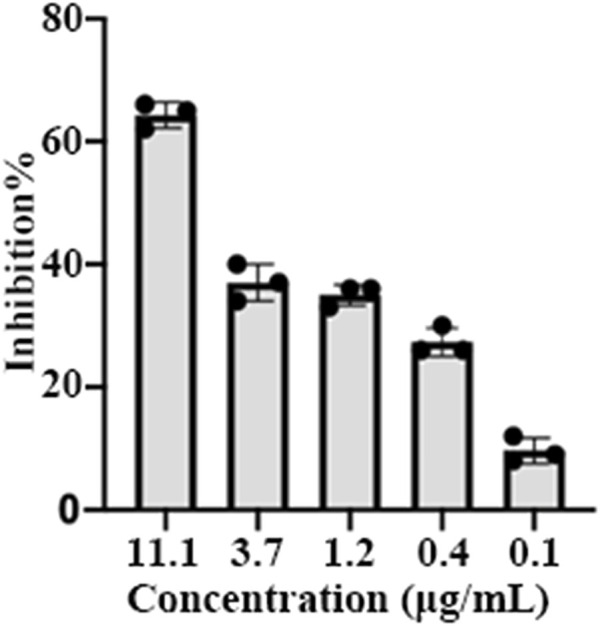
Inhibition of pseudovirus by EPSs from *H*. *hispanica*. Pseudoviruses of SARS-CoV-2 were incubated with 3-fold serially diluted EPSs for 1 h and then transferred into pre-plated Vero E6 cells, followed by incubation for 24 h. Finally, the cells expressing GFP were measured by using a flow cytometer after lysed by trypsin.

To investigate the anti-SARS-CoV-2 activity of EPSs, the inhibition effects of EPSs were determined using pseudovirus in Vero cells. The results showed EPSs from *H*. *hispanica* can efficiently inhibit infection of pseudovirus to Vero E6 cells. The inhibition rate of EPSs toward pseudovirus reached 65% at 11.1 μg/mL (Figure 3).

### Evaluation of the Anti-SARS-CoV-2 Activity of Exopolysaccharides From ***△**HAH_1206*

**FIGURE 4:**
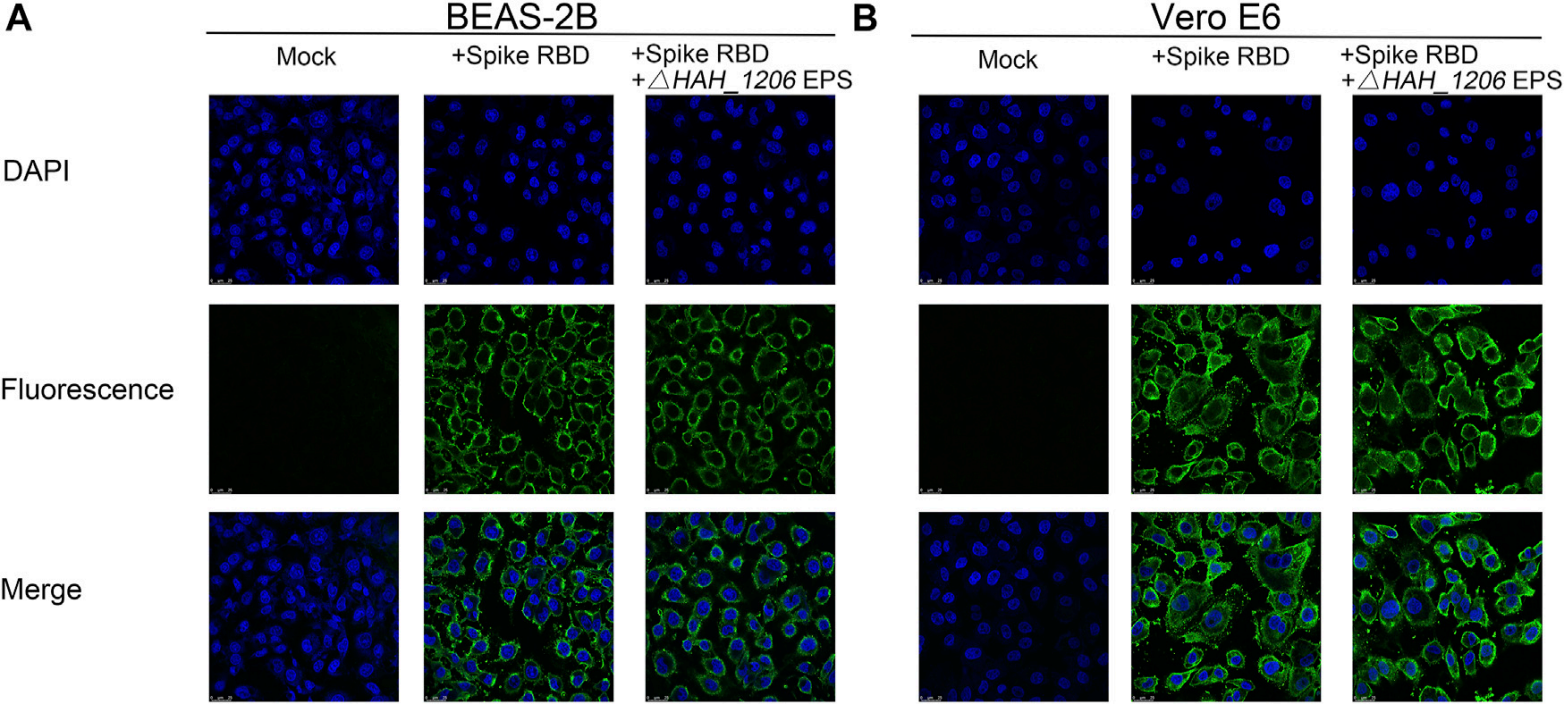
Immunofluorescence assay of EPSs from *△HAH_1206* toward binding of RBD to BEAS-2B and Vero E6. **(A)** Influence of EPSs from *△HAH_1206* on binding of RBD to BEAS-2B cells. **(B)** Influence of EPSs from *△HAH_1206* on binding of RBD to Vero E6. **Mock**: cells detected in the absence of RBD and EPSs; + **Spike RBD**: cells detected in the presence of RBD (1 μg/well); and **+ Spike RBD** + **EPSs from** *△* ***HAH_1206***: cells detected in the presence of RBD (1 μg/well) and EPSs (2 μg/well). All wells were detected by immunofluorescence using SARS-CoV-2 (2019-nCoV) spike-neutralizing antibody and Alexa Fluor 488 goat anti-mouse IgG (H + L) by using a confocal microscope. The fluorescence signals were captured with an FITC channel, and the nuclear DNAs of cell were stained with DAPI.

EPSs from *H*. *hispanica* are the first identified anti-SARS-CoV-2 polysaccharides from archaea, and their antiviral mechanisms are worth investigating. A serial of mutants related to glycosylation in *H*. *hispanica* were constructed and tested (Lu et al., 2020). Compared to the wild-type of *H*. *hispanica*, the *△HAH_1206* mutant was significantly affected (data not shown). Interestingly, it was found that EPSs from *△HAH_1206* could not bind with RBD (Supplementary Figure S3) and did not inhibit the binding of RBD to BEAS-2B and Vero E6 cells either (Figure 4). The results indicated that EPSs from *△HAH_1206* almost completely lost anti-SARS-CoV-2 activity.

### Structural Comparison of Exopolysaccharides From Wild-Type *H*. *hispanica* and ***△**HAH_1206*

**FIGURE 5:**
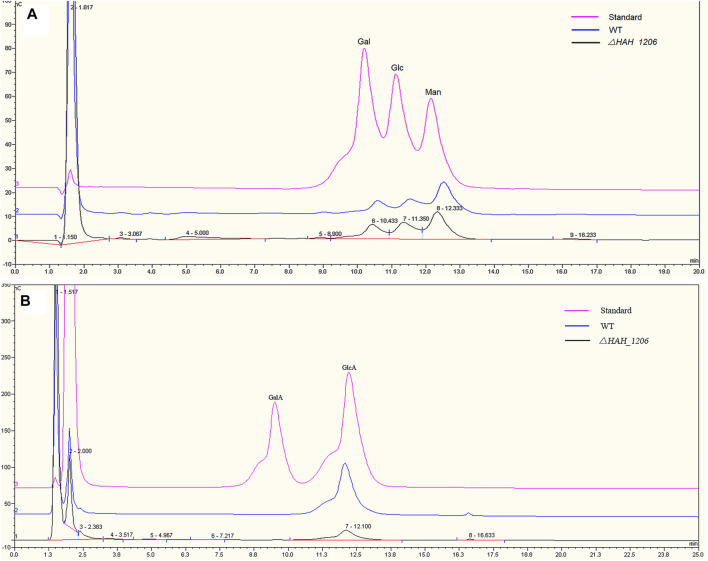
Comparative analysis of monosaccharides in EPSs from *H. hispanica* and *△HAH_1206* with HPAEC-PAD. **(A)** Analysis of neutral monosaccharides of EPSs. The chromatomap in pink is the standard mixture of galactose (Gal), glucose (Glc), and mannose (Man) at 2 mM, whereas the one in blue refers to the hydrolyzed product of EPSs from *H. hispanica* and the black one is the hydrolyzed product of EPSs from *△HAH_1206*. **(B)** Analysis of acidic monosaccharides of EPSs. The chromatomap in pink is the standard mixture of D-galacturonic acid (GalA) and D-glucuronic acid (GlcA) at 2 mM, whereas the blue one represents the hydrolyzed product of EPSs from *H*. *hispanica*, and the black one is the hydrolyzed product of EPSs from *△HAH_1206*.

**FIGURE 6:**
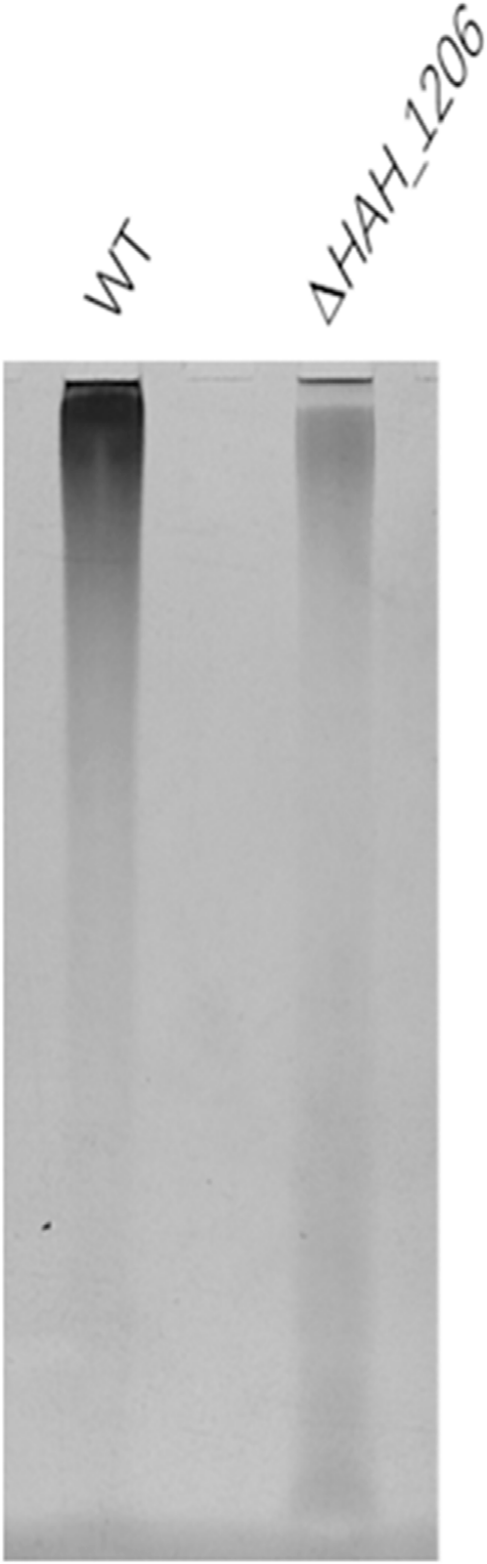
Detection of sulfate in EPSs using the methyl blue method. EPSs (10 μg) from wild-type *H*. *hispanica* and *△HAH_1206* were analyzed in 7.5% (w/v) SDS-PAGE, and sulfates in EPSs were stained with methyl blue.

To investigate the reasons underlining the difference in antiviral activities of EPSs from wild-type *H*. *hispanica* and *△HAH_1206*, their molecular weights (MW), monosaccharide compositions, and sulfation levels were analyzed. The results from HPGPC showed that the MW of EPSs from WT and *△HAH_1206* were 2.126 × 10^7^ Da and 2.007 × 10^7^ Da, respectively, and their MW were nearly identical (Supplementary Figure S4). The sugar compositions were analyzed by HPAEC-PAD. The molar ratio of monosaccharides in EPSs from wild-type *H*. *hispanica* was GlcA:Man:Glc:Gal = 4.3:3.9:1.6:1, and that for *△HAH_1206* was GlcA:Man:Glc:Gal = 0.8:3:1.6:1. These two kinds of EPSs have the same molar ratio of glucose and galactose, and their molar ratios of mannose were slightly different. However, the contents of glucuronic acid in EPSs between two strains were quite different. GlcA in EPSs of *△HAH_1206* was only one-fifth of the wild-type (Figure 5). A significant reduction of GlcA in *△HAH_1206* was clearly observed, suggesting that GlcA might play critical roles in resistance against SARS-CoV-2. Because some sulfated polysaccharides displayed anti-SARS-CoV-2 activity (Kwon et al., 2020; Song et al., 2020), and EPSs from *H*. *hispanica* were also sulfated, the sulfation levels of EPSs from wild-type *H*. *hispanica* and *△HAH_1206* were also compared. An apparent reduction of the sulfation level was found in EPSs from *△HAH_1206* (Figure 6). Combined results of changes in monosaccharide composition and the sulfation level of EPSs from *△HAH_1206* meant GlcA of EPS from wild-type *H*. *hispanica* was heavily sulfated, which was essential for the anti-SARS-CoV-2 activity, and the decline of the sulfation level in *△HAH_1206* might result from reduction of GlcA.

## Discussion

Glycosylation is essential for assembly, recognition, and entry of SARS-CoV-2. The glycosylation sites of N165 and N234 in S protein are important for maintaining the “open” state, and these sites are also necessary for the binding of S protein with hACE2 (Casalino et al., 2020). In addition, the N90, N322, and N546 glycosylation sites in hACE2 can promote the binding of S protein with hACE2 (Zhao et al., 2020). These observations promoted scientists in the field of glycoscience to wonder whether there is a glycan recognition mechanism between SARS-CoV-2 and host cells. Accordingly, they are striving to find an answer and searching for glycan inhibitors against SARS-CoV-2 infection. Recent study showed the spike protein of SARS-CoV-2 cannot recognize sialic acid, but can specifically bind with HS in a sulfation-dependent manner (Hao et al., 2021). Furthermore, the spike protein can bind with hACE2 and HS through different domains, and HS on the surface of host cells can promote the entry of SARS-CoV-2 into host, which can be inhibited by exogenous heparin (Clausen et al., 2020). Moreover, researchers found other sulfated polysaccharides were also potential candidates against SARS-CoV-2, such as fucoidan, carrageenan, and sulfated polysaccharide from sea cucumber. In addition, β-chitosan, xylitol, capsular polysaccharides from *S*. *pneumoniae*, and LPS from *P*. *aeruginosa* can also bind with S protein (Alitongbieke et al., 2020; Chiodo et al., 2020; Song et al., 2020; Bansal et al., 2021). These examples clearly demonstrated that glycans with different structural characteristics could prevent SARS-CoV-2 infection, possibly using different mechanisms.

Archaea are one of the most mysterious parts of global ecosystem, which have developed various adaptations under extreme conditions, and the interaction between archaea and virus is less understood. Therefore, our understanding of antiviral activities of archaea far lags behind our knowledge of those in bacteria, mammals, and plants. Currently, more than 100 archaea have been discovered, and over 90 viruses were described as halophilic archaeal viruses (Atanasova et al., 2015; Snyder et al., 2015). Members of the family *Pleolipoviridae* (termed pleolipoviruses) belong to pseudo-spherical and pleomorphic archaeal viruses, which have a narrow host range as extremely halophilic archaea in the class *Halobacteria* (Bamford et al., 2017). EPSs are biomacromolecules with high molecular weights secreted by microbes. Most of EPSs are heteropolysaccharides containing three to four types of monosaccharides arranged in groups of 10 to form the repeating units (Poli et al., 2010). EPSs around cells can provide effective protection against severe environment and pathogen. Moreover, EPSs have displayed multi-function as anti-microbe, immunomodulator, anti-inflammation, antioxidant, anticancer, hypocholestrolemia, antidiabetes, and antivirus (Angelin and Kavitha, 2020; Abdalla et al., 2021). In this study, EPSs of *H*. *hispanica* can bind to SARS-CoV-2 RBD of spike protein with a high affinity of K_D_ as 2.23 nM and can inhibit the binding of RBD to BEAS-2B and Vero E6 cells. Importantly, the infection of SARS-CoV-2 pseudovirus to Vero E6 cells was effectively blocked by EPSs from *H*. *hispanica*. This is the first report that glycans from archaea can inhibit the infection of SARS-CoV-2.

Another important finding is the anti-SARS-CoV-2 activity of EPSs from *H*. *hispanica* is possibly related to sulfated GlcA. This means sulfation on GlcA may play critical roles in EPSs of *H*. *hispanica* against SARS-CoV-2. As we know, GlcA is the basic monosaccharide of glycosaminoglycans (GAGs), which are ubiquitously present on almost all mammalian cells and considered to be the first interface between a host cell and various bacterial, parasitic, and viral pathogens. GAGs and their derivatives, some of which lack significant anticoagulant activity, are under-exploited antiviral candidate drugs as they possess broad-spectrum activity against a multitude of distinct viruses (Mycroft-West et al., 2020). The repeating disaccharide units of GAGs, comprising a hexosamine and uronic acid or a galactose residue, are often sulfated. The anti-SARS-CoV-2 activities of GAGs have been confirmed in previous reports, along with other sulfated polysaccharides. Therefore, many people presumed that this kind of antiviral activity was possibly related to sulfation (Clausen et al., 2020; Kwon et al., 2020; Song et al., 2020; Hao et al., 2021). However, the exact structural unit of sulfated GAG, which contributes to their anti-SARS-CoV-2 activities, is still unknown. It is likely that the role of sulfated uronic acid in GAGs in anti-SARS-CoV-2 is similar to that of sulfated GlcA in EPSs of *H*. *hispanica*. The current study identified sulfated GlcA in EPSs is important for their anti-SARS-CoV-2 activity, which prompted us to propose that sulfated GlcA in HS is an important structural unit for their anti-SARS-CoV-2 activity. For EPSs biosynthesis in *H*. *hispanica*, a polysaccharide biosynthesis gene cluster has been annotated (Liu et al., 2011), which contained seven genes from *HAH_1661* to *HAH_1667*. The biosynthesis pathway of EPSs in *H. hispanica* has not been fully elucidated, and the details of the process are still less understood. Because mannose is the major composition of EPSs, two genes, *HAH_1662* and *HAH_1667*, which were considered coding for mannosyltransferase were deleted in *H. hipanica* respectively, the mutants of *△HAH_1662* and *△HAH_1667* almost lost acidic EPSs. To confirm the structure and modification of EPSs, we need to characterize the function of each gene in the polysaccharide biosynthesis gene cluster of *H*. *hispanica* in future.

This study identified that sulfated GlcA in EPSs is important for the anti-SARS-CoV-2 activity, which has deepened our understanding of the key structural unit of glycans containing sulfated GlcA toward the anti-SARS-CoV-2 activity. Nowadays, the study about antiviral mechanisms of archaea focused on the nucleic acid level, including the CRISPR-Cas system and DNA phosphorothioation. The unusual metabolic pathways of archaeal cells can produce unique biomacromolecules and metabolites with novel characteristics. EPSs from extremophiles are quite different in composition and characteristics from those in other microbes. EPSs from most mesophilic microbes are toxic, whereas extremophilic microorganisms produce non-pathogenic EPSs, which can be applied in food, pharmaceutical, and cosmetic industries (Nicolaus et al., 2010). Although EPSs from *H*. *hispanica* displayed slight toxicity to cells, they are still good candidates to be developed into antiviral reagents, which would provide a new strategy against SARS-CoV-2.

## Conclusion

In this study, we found that EPSs from halophilic archaeon *Haloarcula hispanica* displayed activities against SARS-CoV-2; it is the first discovery that EPSs from archaea can effectively inhibit SARS-CoV-2 *in vitro*. Compared to EPSs from deletion mutants of *△HAH_1206*, which lost anti-SARS-CoV-2 activity, it is likely that sulfated GlcA in EPSs from wild-type *H. hispanica* contribute to anti-SARS-CoV-2 activities. Our findings will provide a novel antiviral mechanism and a guide for designing new agents against SARS-CoV-2.

## Data Availability

The original contributions presented in the study are included in the article/Supplementary Material, and further inquiries can be directed to the corresponding authors.
