# Supplementary material for: Novel Insights Into the Sulfated Glucuronic Acid-Based Anti-SARS-CoV-2 Mechanism of Exopolysaccharides From Halophilic Archaeon Haloarcula hispanica
Source: Front Chem. 2022 Apr 27;10:871509. doi: 10.3389/fchem.2022.871509 (PMC9091367; doi:10.3389/fchem.2022.871509)
Supplement: Supplementary file 1 [file DataSheet1.docx]

**SUPPLEMENTARY MATERIAL**

**Novel Insights Into the Sulfated Glucuronic Acid-Based Anti-SARS-CoV-2 Mechanism of Exopolysaccharides From Halophilic Archaeaon *Haloarcula hispanica***

*Yueqiang Xu^1†^, Yan Li^2†^, Xin You^3†^, Caixia Pei^4†^, Zhuo Wang^1^, Siming Jiao^1^, Xin Zhao^2^, Xuan Lin^1^, Yang Lü^4^, Cheng Jin^4^, George Fu Gao^2^, Jianjun Li^1*^, Qi Wang^2*^ and Yuguang Du^1*^*

*^1^ State Key Laboratory of* *Biochemical Engineering, National Engineering Research Center for Biotechnology (Beijing), Key Laboratory of Biopharmaceutical Production & Formulation Engineering, PLA, Institute of Processing and Engineering, Chinese Academy of Sciences, Beijing, China; ^2^ CAS Key Laboratory of Pathogenic Microbiology and Immunology, Institute of Microbiology, Chinese Academy of Sciences, Beijing, China; ^3^ Lung Cancer Translational Medicine Center, The Second Affiliated Hospital of Dalian Medical University, Dalian, China; ^4^ State Key Laboratory of Mycology, Institute of Microbiology, Chinese Academy of Sciences, Beijing, China.*

^1^ These authors contributed equally to this work.

* Corresponding authors.


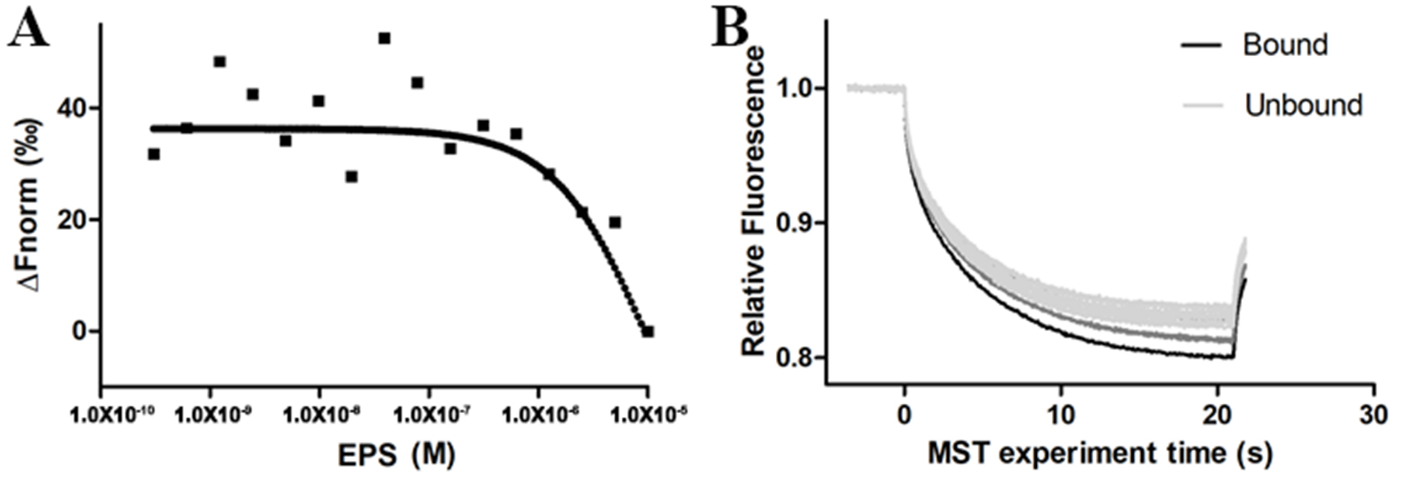


**FIGURE S1. Binding of EPS from *H. hispanica* with RBD by MicroScale Thermophoresis (MST).** The 2-fold-serially diluted EPS solutions were incubated with Monolith RED-NHS labelled RBD, and the binding affinity was measured with Monolith NT.115 (Nanotemper). A: Binding curve of the serially diluted EPS and RBD; B. Trace of binding curve derived from different concentrations of EPS and RBD. Black lines represent the concentrations of bound EPS, while gray lines represent the unbound ones.


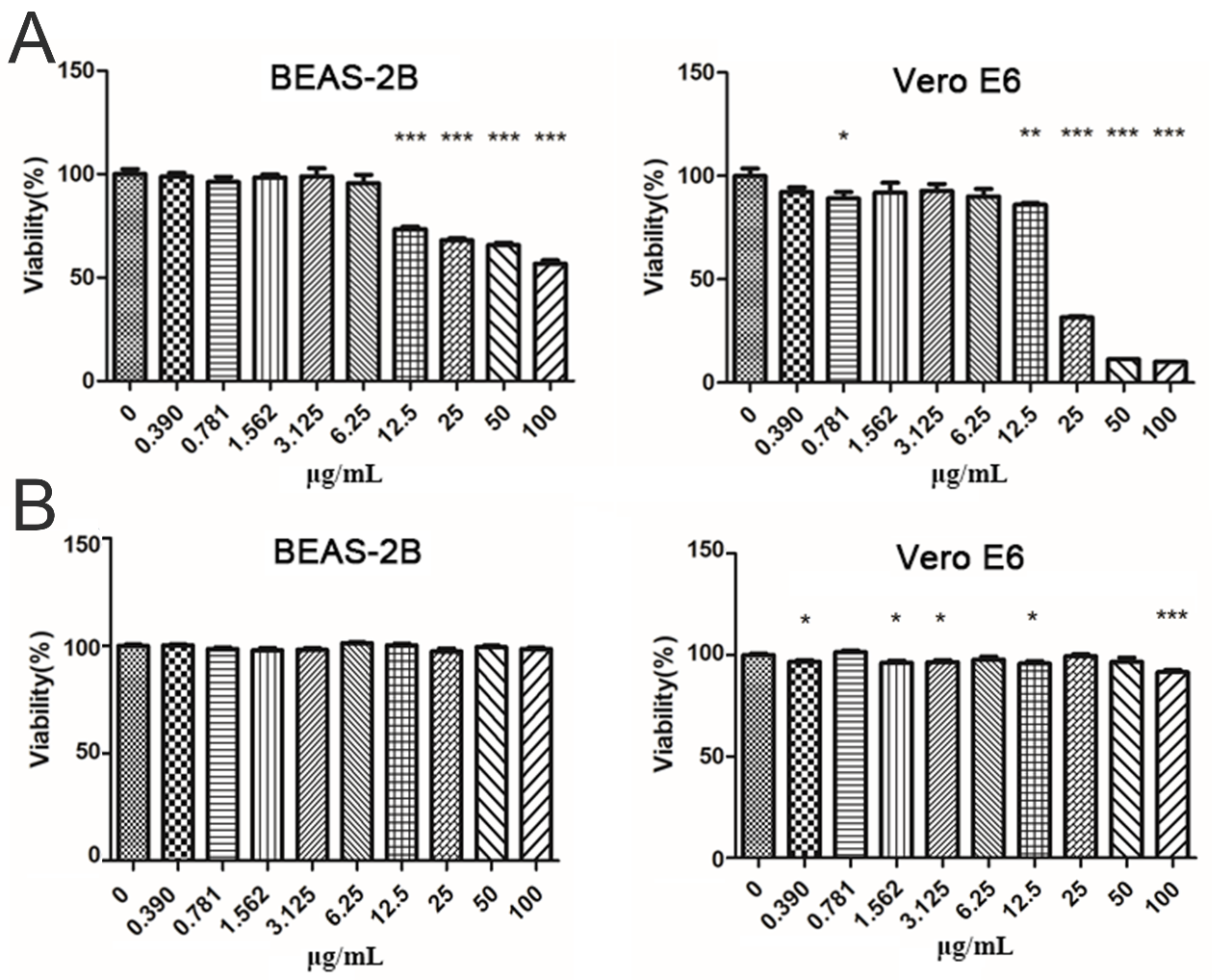


**FIGURE S2. Cell viability of BEAS-2B and Vero E6 cells against EPS from wild-type *H. hispanica* and *△HAH_1206*.** A. Cell viability of BEAS-2B and Vero E6 cells against EPS from wild-type *H.hispanica*, EPS was serially diluted with 2-fold-diluted method from 100 μg/mL to 0.39 μg/mL. B. Cell viability of BEAS-2B and Vero E6 cells against EPS from *△HAH_1206*, EPS was serially diluted with 2-fold-diluted method from 100 μg/mL to 0.39 μg/mL. The results were analyzed by unpaired *t* test with GraphPad.


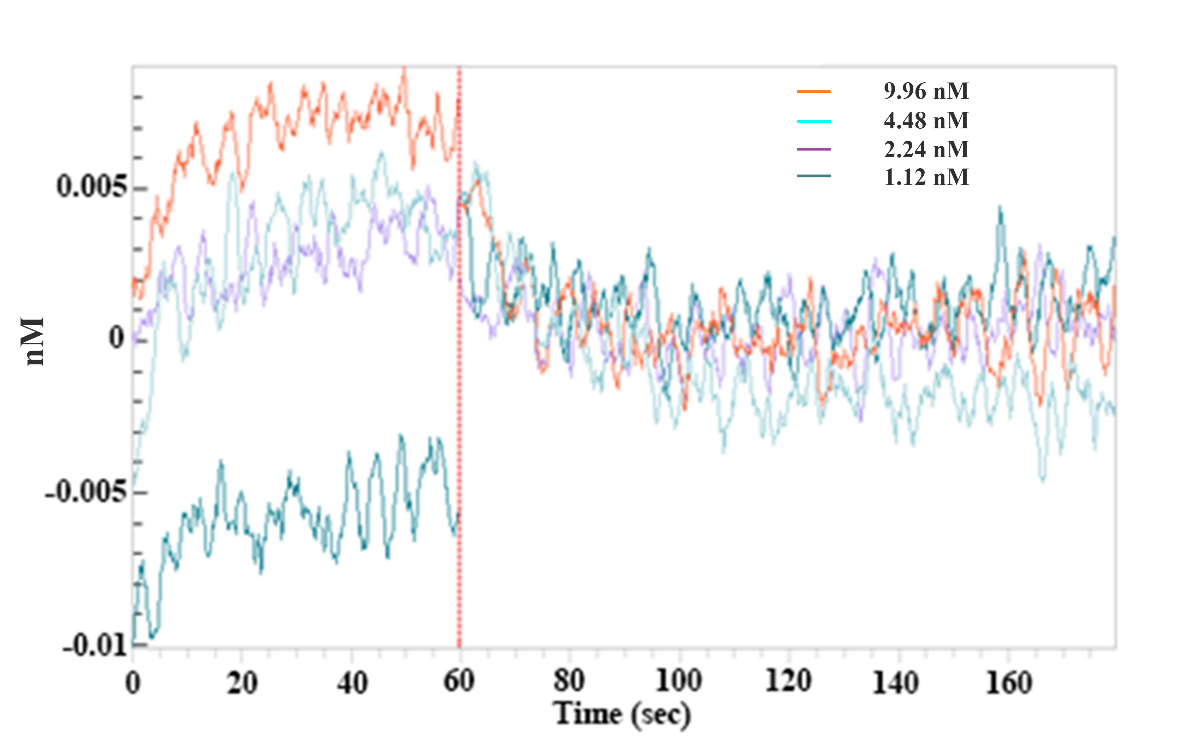


**FIGURE S3. Binding assay between RBD and EPS from *△HAH_1206*.** Serially diluted EPS solutions (9.96 nM to 1.12 nM) were incubated with RBD which was coated on Ni-NTA sensor for 2 minutes respectively, and the affinity kinetics were analyzed after dissociated for 2 min. EPS from *△HAH_1206* completely lost binding affinity to RBD.


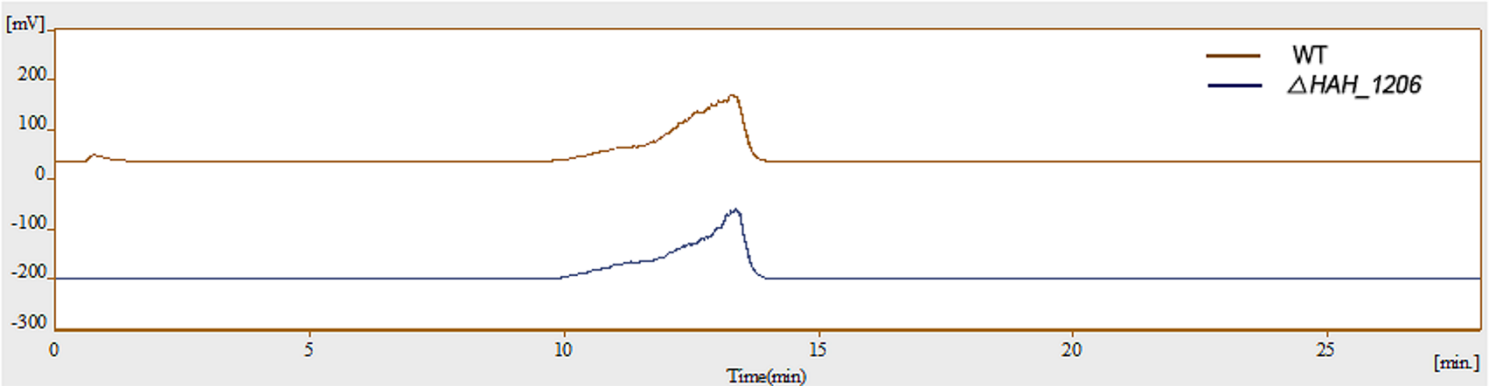


**FIGURE S4. Molecular weight determination of EPS from wild-type *H. hispanica* and *△HAH_1206*.** EPS from wild-type *H.hispanica* and *△HAH_1206* were analyzed by High Performance Gel Permeation Chromatography (HPGPC), the MW for EPS of wild-type *H.hispanica* is 2.126 x 10^7^ (brown), and MW for EPS of *△HAH_1206* is 2.007 x 10^7^ (blue).
